# Phenotypic and genotypic assessment of fluoroquinolones and aminoglycosides resistances in *Pseudomonas aeruginosa* collected from Minia hospitals, Egypt during COVID-19 pandemic

**DOI:** 10.1186/s12879-024-09605-5

**Published:** 2024-07-31

**Authors:** Maria Refaat Boushra, Gamal Fadl Mahmoud Gad, Noha Anwar Hassuna, Nancy Gamil Fawzy Waly, Reham Ali Ibrahem

**Affiliations:** 1 Department of Microbiology and Immunology, Faculty of Pharmacy, Minia University, Minia, Egypt; 2 Department of Medical Microbiology and Immunology, Faculty of Medicine, Minia University, Minia, Egypt

**Keywords:** *Pseudomonas aeruginosa*, Fluoroquinolones resistance, Aminoglycosides resistance, Real-time PCR, RAPD typing

## Abstract

**Background:**

One of the most prevalent bacteria that cause nosocomial infections is* Pseudomonas aeruginosa.* Fluoroquinolones (FQ) and aminoglycosides are vital antipseudomonal drugs, but resistance is increasingly prevalent. The study sought to investigate the diverse mechanisms underlying FQ and aminoglycoside resistance in various *P. aeruginosa* strains particularly during the COVID-19 crisis.

**Methods:**

From various clinical and environmental samples, 110 *P. aeruginosa* isolates were identified and their susceptibility to several antibiotic classes was evaluated. Molecular techniques were used to track target gene mutations, the presence of genes encoding for quinolone resistance, modifying enzymes for aminoglycosides and resistance methyltransferase (RMT). Efflux pump role was assessed phenotypically and genotypically. Random amplified polymorphic DNA (RAPD) analysis was used to measure clonal diversity.

**Results:**

*QnrS* was the most frequently encountered quinolone resistance gene (37.5%) followed by *qnrA* (31.2%) *and qnrD* (25%). Among aminoglycoside resistant isolates, 94.1% harbored modifying enzymes genes, while RMT genes were found in 55.9% of isolates. The *aac(6')-Ib* and *rmtB* were the most prevalent genes (79.4% and 32.3%, respectively). Most FQ resistant isolates overexpressed *mexA* (87.5%). RAPD fingerprinting showed 63.2% polymorphism.

**Conclusions:**

Aminoglycosides and FQ resistance observed in this study was attributed to several mechanisms with the potential for cross-contamination existence so, strict infection control practices are crucial.

## Background

*Pseudomonas aeruginosa* (*P. aeruginosa*) is a ubiquitous Gram-negative microorganism that can flourish in a variety of etiological niches based on its metabolic versatility. Additionally, it is an opportunistic pathogen implicated in an expanding variety of difficult to treat infections, especially in individuals with impaired immune systems [1–3], such as pneumonia linked to ventilator use, infections at the surgical site, urinary tract infections, bacteremia and infection of individuals with thermal injuries [4–6].

One of the most characteristic features of *P. aeruginosa*, which is itself intrinsically resistant to a variety of antimicrobial drugs, is its capability to acquire resistance to available antibiotics. This resistance is expected to increase due to the excessive antibiotic usage during COVID-19 pandemic, increasing the incidence of extensively drug-resistant (XDR) and multidrug-resistant (MDR) clones globally [7, 8]. In 2020, the National Healthcare Safety Network in Georgia stated high antibiotic resistance rates among *P. aeruginosa* infections in healthcare facilities. These rates included cephalosporins (10.2%–30.6%), carbapenems (9.1%–42.6%), fluoroquinolones (11–53.2%), aminoglycosides (5.8%–22.6%), and piperacillin/tazobactam (7.7%–23.9%) [9], increasing morbidity, mortality, duration of hospitalization and treatment expenses [10].

Significant data indicates that the major causes of FQs resistance are i: mutations in quinolone-resistance determining regions (QRDRs), such as *gyrA*/*gyrB* in DNA gyrase and *parC****/****parE* in topoisomerase IV, ii: increased expression of resistance–nodulation–division (RND) efflux systems, especially MexAB-OprM, MexCD-OprJ, MexEF-OprN, and MexXY-OprM, which decrease the drug accumulation inside the cell [11, 12], and iii) the existence of plasmid-mediated quinolone resistance (PMQR) determinants including those belonging to the families of AAC(6′)-Ib-cr, Qnr,, QepA, OqxAB [13].

Likewise,* P. aeruginosa* uses a variety of resistance mechanisms against aminoglycosides such as reduced drug absorption due to restricted membrane permeability, overexpression of MexXY-OprM efflux system, modification of the ribosomal binding site and acquisition of aminoglycoside-modifying enzymes (AMEs) [14]. Furthermore, *P. aeruginosa* strains obtained from clinical samples exhibit additional pathway for aminoglycoside resistance through 16S rRNA methylases [15]. The AMEs are classified as aminoglycoside acetyltransferases (AACs), aminoglycoside phosphotransferases (APHs), and aminoglycoside nucleotidyltransferases (ANTs) [16]. High gentamicin resistance is clearly correlated with *aac(6′)-Ib* gene, that is found in most of Gram-negative bacteria, including *P. aeruginosa* [15, 17]. Pan-resistant *P. aeruginosa* was used to study other common AMEs encoded by *aph(3′)-VI, ant(3″)-I, aac(6′)-II aac(3′)-II and ArmA* [18].

Inhibitors have been used to stop bacterial efflux pumps from working; one of the most significant inhibitors in this context is carbonyl cyanide 3-chlorophenyl hydrazone (CCCP). This RND family inhibitor enhances the concentration of antibiotics inside the bacterial cell, increasing the drug's potency by interfering with the efflux pump's activity [19].

The purpose of the work was to identify and functionally characterize the different mechanisms underlying FQ and aminoglycosides resistance in environmental and clinical* P. aeruginosa* isolates, especially during COVID-19 crisis. We focused specifically on these two classes of antibiotics because *P. aeruginosa* infections are frequently treated with them. As far as we are aware, our study is the first to investigate *Pseudomonas aeruginosa* resistance to aminoglycosides and fluoroquinolones in samples taken from Minia University during the COVID-19 outbreak.

## Methods

### *P. aeruginosa* isolation and identification

The study was carried out at the Minia Hospitals from July 2020 to August 2021 during the COVID-19 pandemic. A total of 104 *P. aeruginosa* isolates were recovered from several clinical specimens (21 wound, 20 ear discharge, 16 sputum, 15 urine, 11 stool, 9 tracheal aspirates, 8 blood and 4 eye swabs) collected from patients suffering from various infections as a part of the hospital ordinary laboratory testing. Additionally, 6* P. aeruginosa* isolates were obtained from random environmental surfaces samples (beds, walls, devises and table tops). Ethical approval for the study procedures was granted by the ethics committee of faculty of Pharmacy, Minia University (No. 230603). Isolates were identified using traditional laboratory techniques [20]. For additional testing, isolates were kept for an extended period of time at -80 °C in a 25% glycerol stock [21]. The reference strain utilized during the study was *P. aeruginosa* ATCC 27853.

## Antimicrobial susceptibility testing

The antibiotic susceptibility pattern of all isolated *P. aeruginosa* was assessed using the Kirby-Bauer disc diffusion technique on Mueller–Hinton agar (MHA) (HiMedia, India) following the criteria provided by the Clinical and Laboratory Standards Institute (CLSI) [22], the tested antibiotics were piperacillin/tazobactam (100/10 μg), cefepime (30 µg), ceftazidime (30 µg), aztreonam (30 μg), meropenem (10 μg), imipenem (10 μg), ciprofloxacin (5 μg), levofloxacin (5 μg), norfloxacin (10 μg), ofloxacin (5 μg), lomefloxacin (10 μg), gatifloxacin (5 μg), amikacin (30 μg), gentamicin (10 μg) and tobramycin (10 μg) (Oxoid, Basinstoke, UK). Utilizing the agar dilution technique on MHA, the minimum inhibitory concentration (MIC) of colistin sulphate (Sigma-Aldrich, St. Louis, Mo., USA) was determined. Resistance phenotypes were categorized as follows: pan drug resistant (PDR) when the isolate isn't sensitive to all agents in all antibiotic groups; an XDR when the isolate remains sensitive to drug(s) in just one or two antibiotic groups; and MDR when the isolate isn't sensitive to minimally one drug in three or more antibiotic groups [23].

## Evaluation of efflux pump activity using inhibitor based method

To investigate the existence of efflux pump mechanism phenotypically, MICs of ciprofloxacin, levofloxacin, norfloxacin, ofloxacin, gentamicin, tobramycin and amikacin (EPICO, Egypt) against FQ and aminoglycosides resistant isolates were further determined using agar dilution method alone and in combination with CCCP (Sigma-Aldrich, St. Louis, Mo, USA). On MHA plates, the final CCCP concentration was 6.25 μg/ml, calculated to be lower than all MIC values of CCCP against our isolates to exclude the bactericidal effects of the EPI. At least a four-fold reduction in each antibiotic's MIC after CCCP insertion was indicative for the existence of active efflux pumps [24].

## Detection of fluoroquinolones and aminoglycosides resistance genes

Genomic DNA from All FQ and aminoglycosides resistant isolates was extracted by boiling procedures previously described by Adabi co-workers [25] and kept at-20 °C before using as a template in PCR amplification.

Conventional PCR was used to detect the prevalence of aminoglycoside resistance genes (*aac(6’)-Ib aac(3)-Ia, ant(2″)-Ia, ant(4’)-IIa, aph(3″)-Ib, armA, rmtB, rmtC and rmtD*) and PMQR genes, namely *qnrS, qnrA, qnrB, qnrD* and *qepA*, among resistant *P. aeruginosa* isolates. PCR primers and specific cycling conditions are listed in Table 1.

**Table 1:** Primers used in this study and specific conditions for conventional and real-time PCR reactions

Primer		Sequence (5ʹ-3ʹ)	Annealing temperature	Size (bp)	References
**PMQR genes**	*qnrA*	F: AGAGGATTTCTCACGCCAGG R:TGCCAGGCACAGATCTTGAC	55^∘^C	580	[26]
	*qnrB*	F: GGCATTGAAATTCGCCACTG R: TTTGCTGCTCGCCAGTCGAA	55^∘^C	263	[26]
	*qnrD*	F: CGAGATCAATTTACGGGGAATA R: AACAAGCTGAAGCGCCTG	56^∘^C	533	[26]
	*qnrs*	F: ACGACATTCGTCAACTGCAA R: TAAATTGGCACCCTGTAGGC	54^∘^C	417	[27]
	*qepA*	F: AACTGCTTGAGCCCGTAGAT R: GTCTACGCCATGGACCTCAC	55^∘^C	596	[26]
**AMEs genes**	*aac(6’)-Ib*	F: TTGCGATGCTCTATGAGTGGCTA R: CTCGAATGCCTGGCG TGT TT	55^∘^C	472	[16]
	*aac(3)-Ia*	F: GGCTCAAGTATGGGCATCAT R: TCACCGTAATCTGCTTGCAC	52^∘^C	389	[27]
	*ant(4’)-IIa*	F: ATCGTCTGCGAGAAGCGTAT R: TAAAACGCCTATCCGTCACC	52^∘^C	839	[27]
	*aph(3″)-Ib*	F: CTTGGTGATAACGGCAATTCC R: CCAATCGCAGATAGAAGGCAA	52^∘^C	548	[27]
	*ant(2″)-Ia*	F: GACACAACGCAGGTCACATT R: CGCAAGACCTCAACCTTTTC	55^∘^C	500	[27]
**RMTs genes**	*armA*	F: ATTCTGCCTATCCTAATTGG R: ACCTATACTTTATCGTCGTC	50^∘^C	315	[16]
	*rmtB*	F: GCTTTCTGCGGGCGATGTAA R: ATGCAATGCCGCGCTCGTAT	50^∘^C	173	[16]
	*rmtC*	F: CGAAGAAGTAACAGCCAAAG R: ATCCCAACATCTCTCCCACT	50^∘^C	711	[16]
	*rmtD*	F: CGGCACGCGATTGGGAAGC R: CGGAAACGATGCGACGAT	50^∘^C	401	[16]
**QRDRs**	*gyrA*	F: AGTCCTATCTCGACTACGCGAT R: AGTCGACGGTTTCCTTTTCCAG	55^∘^C	378	[28]
	*parC*	F: CGAGCAGGCCTATCTGAACTAT R:GAAGGACTTGGGATCGTCCGGA	55^∘^C	304	[28]
**Real time PCR genes**	*mexA*	F: AACCCGAACAACGAGCTG R: ATGGCCTTCTGCTTGACG	55^∘^C		[29, 30]
	*mexC*	F: GGAAGAGCGACAGGAGGC R: CTGCACCGTCAGGCCCTC	55^∘^C		[29, 30]
	*mexE*	F: TACTGGTCCTGAGCGCCT R: CAGCGGTTGTTCGATGA	55^∘^C		[29, 30]
	*mexY*	F: CCGCTACAACGGCTATCCCT R: AGCGGGATCGACCAGCTTTC	55^∘^C		[30, 31]
	*Rpsl*	F: GCAACTATCAACCGACTGGTG R: GCTGTGCTCTTGCAGGTTGTG	55^∘^C		[30, 31]

*Abbreviations: PMQR* plasmid-mediated quinolone resistance, *AMEs* aminoglycoside modifying enzymes, *RMT* resistance methyltransferase, *QRDR* quinolone-resistance determining regions

Three multiplex PCR assays were carried out. The first reaction included the *aac(3)-Ia, ant(4’)-IIa* and *aph(3″)-Ib.* The second reaction involved *armA* and *rmtC,* while the third one included *rmtB* and *rmtD.*

A 25 μl PCR reaction mixture was prepared comprising of 12.5 μl of 2 × PCR ready-to use Master mix (COSMO PCR Hot Start Master Mix, willowfort, UK), 1 μl of each primer (10 pM/µl) (forward and reverse), 2 μl of template DNA and nuclease-free water. The amplified products were examined by 1% w/v agarose gel electrophoresis and observed under UV illumination. Ready-made 100 bp Plus DNA marker (Thermo-scientific™, USA) was utilized to detect the amplicon size.

## PCR amplification and DNA sequencing

The amplification of *gyrA* and *parC* from isolates showed resistance to FQ was performed by conventional PCR using cycling conditions and primer sets described in Table 1. Gel electrophoresis was employed to verify their presence while the size of bands was detected by Primer-Blast driver (www.ncbi.nlm.nih.gov/tools/primer-blast). Purified products were submitted for Sanger sequencing with the same sets of primers, utilizing the Applied Biosystems 3730/3730xl DNA analyzer sequencing (ABI) system (Thermo-Scientific™, USA). The Molecular Evolutionary Genomic Analysis (MEGA11) program was applied for multiple sequences alignment by Clustal W, and to compare it with *P. aeruginosa* ATCC27853 reference sequences to detect mutation [14].

## Extraction of total RNA and qRT-PCR analysis

Resistant isolates of* P. aeruginosa* were cultivated in 1.5 ml Luria–Bertani (LB) broth (HiMedia, India) at 37 °C for 18–24 h, and the GeneJET RNA Purification Kit (Thermo-Scientific™, USA) was used to extract the total RNA from exponentially growing bacteria based on the supplier’s instructions. The cDNA synthesis was performed in a 20 μl total volume following to the producer’s recommendations utilizing Revert Aid First Strand cDNA synthesis Kit (Thermo-Scientific™, USA). The obtained cDNAs were kept at temperature of − 20 °C until use.

The expression of *mexA*, *mexC*, and *mexE* genes in FQ-resistant isolates, as well as *mexY* gene in all FQ and aminoglycoside-resistant isolates, was measured using real-time PCR (RT-PCR) comparing with the housekeeping gene *rpsl.* Table 1 displays the primers used in the amplification of these genes. Using Applied Biosystems 7500-Fast Real-Time PCR system (Thermo-Scientific™-USA), transcripts quantification was performed by SYBRGreen PCR Master Mix (Thermo-Scientific™, USA) in total volume of 10 μl. The ΔΔCt equation has been applied to determine the relative gene expression [32]. The expression of each genes was normalized against the housekeeping gene *rpsl* of the same strain and calibrated in relation to target gene expression in *P. aeruginosa* ATCC27853 [33]. MexAB-OprM, MexCD-OprJ, MexEF-OprN, and MexXY-OprM were deemed to be overproduced when the transcriptional levels of *mexA, mexC, mexE*, or *mexY* were at least 2, 2, 10, or 4 times greater than those of *P. aeruginosa* ATCC27853, respectively [30].

## Random amplified polymorphic DNA (RAPD) PCR

The DNA of isolated *P. aeruginosa,* resistant to fluoroquinolones and aminoglycosides, were subjected to RAPD fingerprinting utilizing primer 272-AGCGGGCCAA manufactured by Mahenthiralingam et al. [34]. PCR amplification was conducted using the reaction mixture components mentioned above in this study. The utilized thermocycler program was reported in a previous study [35]. Electrophoresis on 1% (w/v) agarose gels was used to separate the amplified products using 1 Kb DNA marker (Thermo-Scientific™, USA) in each gel. Using the FreeTree and TreeView software, the similarity between resistant strains was evaluated based on Dice similarity coefficient and Unweighted Average Pair Group Method (UPGMA). For similarity matrix calculation, only main replicable bands were put into consideration. Potential clonal relatedness was determined using cut-off values of ≥ 80% [35].

## Statistical analysis

To analyze the data, Statistical Package of Social Science (SPSS) software version 22 (Chicago, USA) was employed. The following had been performed accordingly: qualitative variables were presented by numbers and percentages, Chi-square and Z score tests for comparison of categorical data, Kruskall-Wallis to compare medians and bivariate Spearman correlation analysis to show degree of association between quantitative variables. *P* values < 0.05 for all tests were regarded as statistically significance. Correlation Grades were determined in the following way: from 0.00 to 0.24 means weak or no association, from 0.25 to 0.49 indicates fair association, from 0.50 to 0.74 points to moderate association and 0.75 + signifies strong association.

## Results

**Fig. 1:**
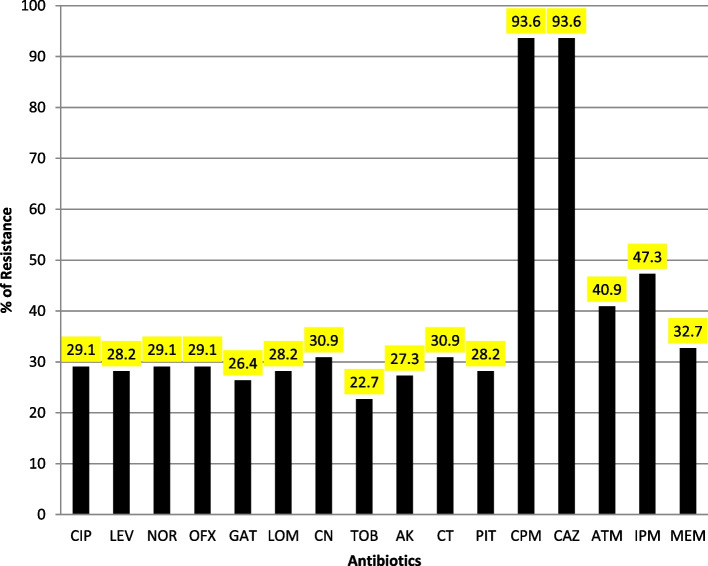
Antimicrobial resistance profile of *P. aeruginosa* isolates. *Abbreviations: CIP* ciprofloxacin, *LEV* levofloxacin, *NOR* norfloxacin, *OFX* ofloxacin, *GAT* gatifloxacin, *LOM* lomefloxacin, *CN* gentamicin, *AK* amikacin, *TOB* tobramycin, *CT* colistin, *PIT* piperacillin-tazobactam, *CPM* cefepime, *CAZ* ceftazidime, *ATM* aztreonam, *IPM* imipenem, *MEM* meropenem

According to the antimicrobial susceptibility testing, tobramycin and gatifloxacin were the most effective antibiotics in this study (Fig. 1). Among all of isolates, 51/110 (46.4%) showed resistance phenotypes, as the following: 27/51 (52.9%) were MDR, 15/51 (29.4%) were XDR and 9/51(17.6%) were PDR**.** Furthermore, it was discovered that out of 110 *P. aeruginosa* isolates, 28 (25.4%) showed resistance to both aminoglycoside and fluoroquinolones (resistant to a minimum of one antibiotic from each tested class), while 6 (5.4%) and 4 (3.6%) isolates were correspondingly resistant to aminoglycoside and FQ each alone. Moreover, 28 (25.4%) and 23(20.9%) isolates showed resistant to all tested FQ and aminoglycoside, respectively.

## Efflux pump inhibitors' impact on antibiotic resistance

The MICs of resistant* P. aeruginosa* isolates against some fluoroquinolones and aminoglycosides antibiotics were compared with and without CCCP to confirm the major role of the efflux pump phenotypically. Table 2 displays the reduced MICs of antibiotics in the presence of CCCP compared to antibiotics alone. The number of resistant strains that showed ≥ fourfold reduction in MIC of tobramycin, amikacin and norfloxacin were found statistically significant comparing with the phenotypically negative isolates (*P* < 0.05).

**Table 2:** Changes in MIC values of the tested antibiotics after CCCP addition

MIC reduction	Isolates tested for MIC change after CCCP addition No. (%*)						
	**GEN** ***n* = 34**	**TOB** ***n* = 25**	**AK** ***n* = 30**	**CIP** ***n* = 32**	**LEV** ***n* = 31**	**NOR** ***n* = 32**	**OFX** ***n* = 32**
**Unchanged**	11(32.4)	17[36]	21[37]	13(40.6)	11(35.5)	17(53.1)	9(28.1)
**twofold**	3(8.8)	3[12]	3[10]	1(3.1)	5(16.1)	4(12.5)	7(21.9)
** ≥ fourfold**	20(58.8)	5[20]	6[20]	18(56.3)	15 (48.4)	11(34.4)	16[38]
***P* value**	0.14	0.0001	0.0001	0.32	0.81	0.01	-

^*^Percent correlated to the total number of resistant isolates of each antibiotic

*Abbreviations: GEN* gentamicin*, TOB* tobramycin, *AK* amikacin, *CIP* ciprofloxacin, *NOR* norfloxacin, *OFX* ofloxacin

## Detection of fluoroquinolones and aminoglycosides resistance genes:

PCR assay was done to determine the frequency of PMQR genes in FQ resistant isolates. Nineteen (59.4%) of the resistant strains exhibited one or more of the evaluated PMQR genes. *The qnrs, qnrA, qnrD, qnrB* and *qepA* genes were found in 12(37.5%), 10(31.2%), 8(25%) and 4, 4(12.5% each) of the 32 isolates, respectively. All tested PMQR resistance genes were present in 3.1% of isolates, while no resistance genes were observed in 40.6% of the isolates (Table 3).

**Table 3:** Distribution of PMQR genes alone and in combination with each other among the resistant *P. aeruginosa* strains

Genes	No. of isolates (%*)	*P* value
*qnrS*	3 (9.4%)	
*qnrA*	3 (9.4%)	
*qnrD*	2 (6.3%)	
*qnrA* + *qnr S*	4(12.5%)	
*qnrB* + *qepA*	1(3.1%)	
*qnrD* + *qnrS*	1(3.1%)	
*qnrA* + *qnrD* + *qnrS*	2(6.3%)	
*qnrB* + *qnrD* + *qepA*	1(3.1%)	
*qnrB* + *qnrD* + *qnrS* + *qep A*	1(3.1%)	
*qnrA* + *qnrB* + *qnrD* + *qnrS* + *qep A*	1(3.1%)	
PMQR genes positive	19 (59.4%)	0.13
PMQR genes negative	13(40.6%)	
Total	32(100%)	

^*^Percent related to the total number of FQ resistant *P. aeruginosa* isolates

Among 34 aminoglycoside resistant isolates, 32(94.1%) were found to carry AMEs- coding genes, while genes coding for RMT were present in 19(55.9%) isolates. Co-occurrence of both resistances mechanisms was encountered in 17 (50%) isolates. PCRtest verified the existence of *aac(6’)-Ib* in 27(79.4%) isolates either alone or in different combination, followed by *aph(3″)-Ib* gene in 23(67.7%) and *aac(3)-Ia* in 12 (35.3%) strains. While, none of the studied strains harbored *ant(4’)-IIa* gene. The results also revealed predominance of *rmtB gene* (32.3%) among RMT encoding genes. The distributions of these enzymes among resistant isolates were described in Table 4.

**Table 4:** Distribution of AMEs and RMT genes among the resistant *P. aeruginosa* strains

Gene		No. (%*)	*P* value
**AMEs genes**	*aac(6’)-Ib*	3(8.8)	
	*aph(3″)-Ib*	3(8.8)	
	*aac(6’)-Ib* + *aph(3″)-Ib*	5(14.7)	
	*aac(6’)-Ib* + *aac(3)-Ia*	5(14.7)	
	*aac(6’)-Ib* + *ant(2″)-Ia*	3(8.8)	
	*aac(3)-Ia* + *aph(3″)-Ib*	2(5.9)	
	*ant(2″)-Ia* + *aph(3″)-Ib*	1(2.9)	
	*aac(6’)-Ib* + *aac(3)-Ia* + *aph(3″)-Ib*	5(14.7)	
	*aac(6’)-Ib* + *ant(2″)-Ia* + *aph(3″)-Ib*	3(8.8)	
	*aac(6’)-Ib* + *aac(3)-Ia* + *ant(2″)-Ia* + *aph(3″)-Ib*	2(5.9)	
	AMEs genes positive	32(94.1%)	0.0001**
	AMEs genes negative	2(5.9)	
	Total	34(100%)	
**RMT genes**	*rmtB*	9(26.5)	
	*rmtD*	4(11.8)	
	*armA*	2 (5.9)	
	*armA* + *rmtD*	2(5.9)	
	*rmt B* + *rmtC*	1(2.9)	
	*rmtA* + *rmtB*	1(2.9)	
	RMT genes positive	19(55.9%)	0.33
	RMT genes negative	15(44.1)	
	Total	34(100%)	

^*^Percent correlated to the total number of aminoglycoside resistant *P. aeruginosa* isolates

^**^P-value < 0.05 is significant

*Abbreviations: AMEs* aminoglycoside modifying enzymes, *RMT* resistance methyltransferase

Regarding the prevalence of each resistance mechanism (PMQR, AMEs and RMT genes) among resistant isolates, statistical data showed a significant difference regarding distribution of aminoglycoside resistance by enzymatic modification among resistant isolates (*P* = 0.0001).

## Amplification and sequencing of *gyrA *and *parC*

The *gyrA* and *parC* genes were identified in all of the tested isolates and no amino acid alterations was discovered when their sequences were compared to those of *P. aeruginosa* ATCC27853.

## Efflux Pump Genes Expression

The result of RT-PCR showed that most of FQ resistant strains showed overexpression for one or more of the tested *mex* genes (31/32, 96.9%). *MexA* was the most frequently overexpressed gene (28/32, 87.5%), followed by *mexC* (11/32, 34.4%), whereas *mexE* (4/32, 12.5%) was the least one. *MexY* were overexpressed in 12/38 (31.5%) of FQ and aminoglycosides resistant strains (Table 5). In addition, 3/32 (9.4%) of FQ resistant isolates showed overexpression for all four tested RND efflux pump genes followed by the combination of *mexA* + *mexC* + *mexY* in 4/32 (12.5%) of the isolates.

**Table 5:** Distribution of efflux pump genes over-expression among fluoroquinolones and aminoglycoside resistant *P. aeruginosa* isolates

Type of isolates	mexA*	mexC*	mexE*	mexY**
Resistant to FQ only	3(9.4%)	1(3.1%)	1(3.1%)	1(2.6%)
Resistant to aminoglycosides only	Not tested	Not tested	Not tested	1(2.6%)
Resistant to both of FQ and aminoglycosides	25(78.1)	10(31.3%)	3(9.4%)	10(26.3%)
Total	28(87.5%)	11(34.4%)	4(12.5%)	12(31.5%)

^*^Percent of related to the number of FQ resistant isolates

^**^Percent related to the total number of FQ and aminoglycosides resistant isolates

Statistical data revealed that, there was no significant difference between *mexY* over-expression level in isolates displaying resistance to both classes of antibiotics (FQ or aminoglycosides) and those showing resistance to only one class of them (Table 6). Table 7 shows non-significant association between *mexY* expression and gentamicin resistance (r = 0.34, *P* = 0.05). Additionally, there was no significant correlation between *mexE, MexC, MexY* genes expression and levofloxacin resistance.

**Table 6:** Difference *mexY* over-expression level among fluoroquinolones and aminoglycoside resistant *P. aeruginosa* isolates

	**In isolates resistant to FQ only**	**In isolates resistant to aminoglycosides only**	**In isolates resistant to both FQ and aminoglycosides**	***P*** **Value**
Median of folds of over-expression of *mexY* gene	44.2	42.85	14.67	0.78

*P*-value < 0.05 is significant

**Table 7:** Bivariate correlation analysis between efflux genes over-expression of and resistance to levofloxacin and gentamicin

Efflux pump		Levofloxacin	Gentamicin
*mexA*	(r) P	-0.14 0.45	Not tested
*mexC*	(r) P	0.09 0.61	Not tested
*mexE*	(r) P	0.09 0.63	Not tested
*mexY*	(r) P	0.05 0.79	0.34 0.05

Significant correlation at *P* value < 0.05

r: Spearman’s correlation

## Agreement between genotypic and phenotypic detection of efflux pump

Comparing both genotypic and phenotypic methods for efflux pump detection, the current study showed that the addition of CCCP resulted in ≥ fourfold reduction in the MIC of one or more of the studied antibiotics in 27 (87.1%) of 31 FQ resistant isolates that exhibited overexpression of efflux pump genes, while the only isolate that showed no overexpression of any of the tested efflux genes was phenotypically positive. Regarding aminoglycosides efflux pump, 10 (45.5%) of 22 *P. aeruginosa* isolates that showed phenotypic efflux pump-overexpressing had increased expressions of efflux pump genes. Whereas out of 12 phenotypically negative *P. aeruginosa* isolates, only 1 isolate (8.3%) exhibited an overexpression of efflux system genes. Statistics showed non-significant difference between the 2 methods in FQ resistant isolates (*P* = 0.7) in contrast to aminoglycosides resistant isolates in which the difference was statistically significant (*P* = 0.03).

## Prevalence of different fluoroquinolones and aminoglycosides resistance mechanisms among *P. aeruginosa* isolates

**Fig. 2:**
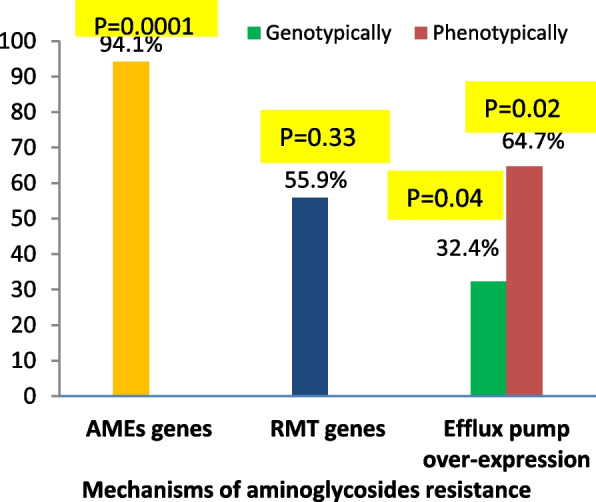
Prevalence of different aminoglycosides resistance mechanisms among resistant *P. aeruginosa* isolates. *Abbreviations: AMEs* aminoglycoside modifying enzymes, *RMT* resistance methyltransferase

**Fig. 3:**
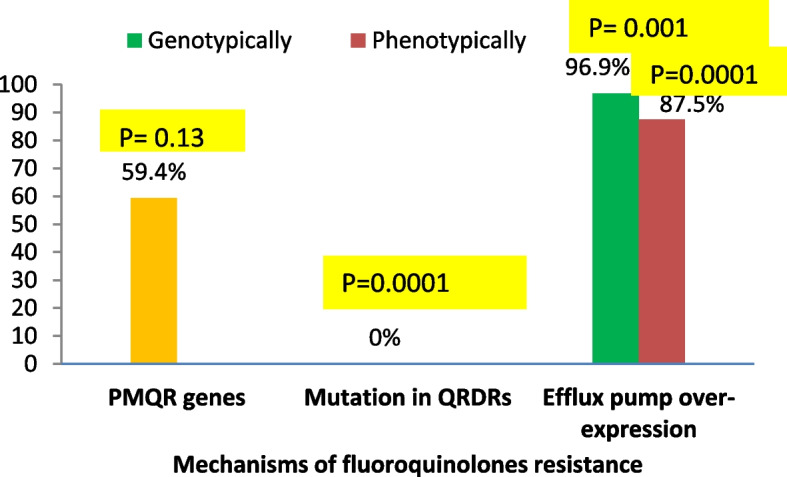
Prevalence of different fluoroquinolones resistance mechanisms among resistant* P. aeruginosa*. Isolates**.** *Abbreviations: PMQR* plasmid-mediated quinolone resistance, *QRDRs* quinolone-resistance determining regions

Figures 2 and 3 display the distribution of each resistance mechanism among the investigated strains of *P.aeruginosa* conferring resistance to aminoglycoside and FQ. Our result revealed that inactivation by AMEs was the main resistance mechanism to aminoglycosides (94.1%), while overexpression of efflux pump genes was the major mechanism for FQ resistance (96.9%). A highly significant difference concerning the distribution of the 2 major resistance mechanisms among FQ and aminoglycoside resistant isolates was detected (*P* = 0.001 and 0.0001 respectively).

## RAPD Genotyping

**Fig. 4:**
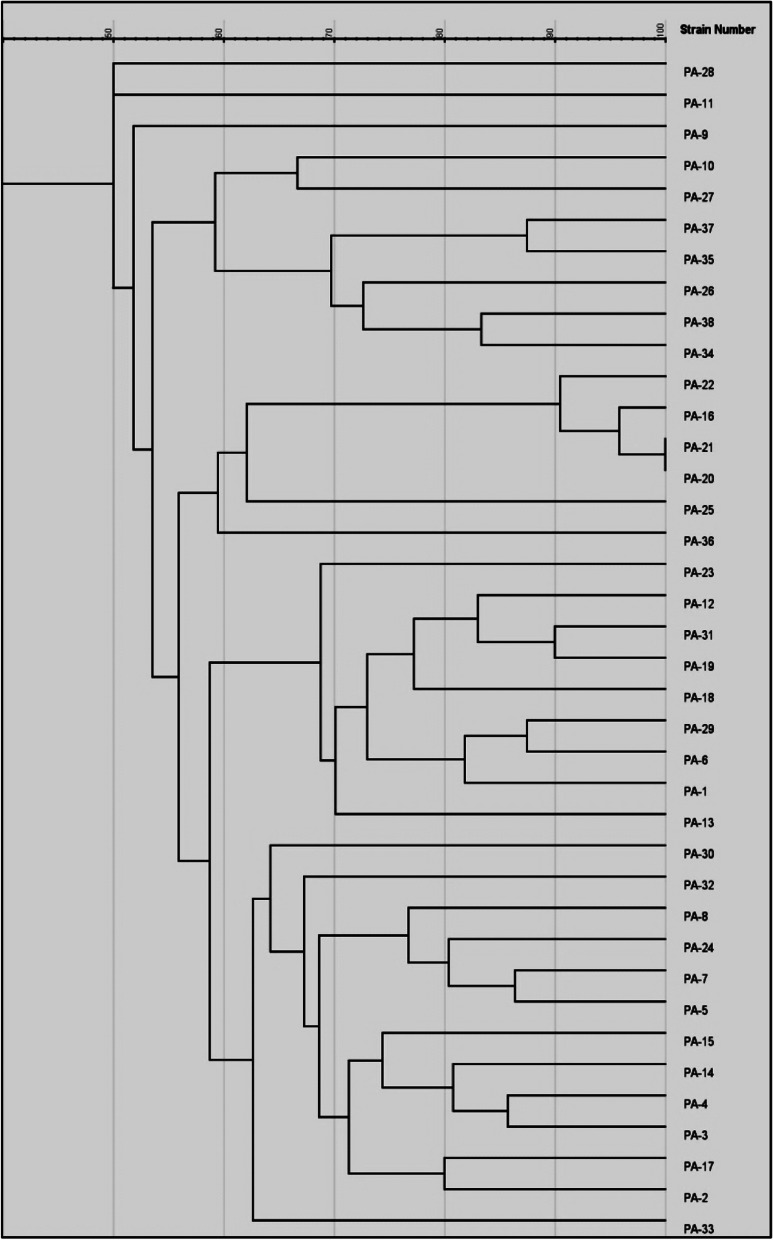
Dendrogram for 38 *P. aeruginosa* strains based on RAPD analysis. The dendrogram was created by gelJ program *Abbreviation: PA Pseudomonas aeruginosa*

A dendrogram was constructed using RAPD data from 38 FQ and aminoglycosides resistant isolates (Fig. 4). RAPD typing generated 6–12 bans with sizes ranging from 250 bp to 3 kb. The study showed high clonal diversity, with 24 different clusters (63.2% of polymorphisms). Sixteen *P. aeruginosa* isolates displayed unique patterns; while the remaining isolates [22] showed 8 distinct clusters of which 2 isolates (PA-20, 21) had 100% similarity.

## Discussion

*P. aeruginosa* represents a main cause of severe infections, associated with an elevated morbidity and mortality rates due to its high levels of antibiotic resistance. In our investigation, 24.5% of total isolates were MDR and 13.6% were XDR. This rate was similar to that obtained previously from Upper Egypt where 22.5% of isolates were MDR [36] and lower than that reported in prior studies [37, 38]. However, it was slightly higher than the frequency of XDR and MDR isolates in Iran (15.5% and 16.5%, respectively) [39].

Regarding the incidence of FQ resistance, 29.1% of the identified isolates were resistant to at least one of the evaluated FQ. This resistance percentage was slightly higher than those found in invasive (cerebrospinal fluid or blood) *P. aeruginosa* strains in Spain (18.1%) and Europe (18.9%) [40], but lower than those of López et al. (37.3%) [13]. Another study from Iran reported high resistance level to ciprofloxacin (74.5%) [41], while our results reported only 29% resistance to ciprofloxacin. Recently, an Egyptian investigation studied the antimicrobial resistant pattern of *P. aeruginosa* during Covid-19 era and revealed higher resistance levels against different antibiotic classes [42].

The total incidence of aminoglycoside resistance detected in this study (30.9%) was less than what other studies had previously reported [43, 44]. Despite this, several investigations revealed substantial rates of amikacin and gentamycin resistance in pseudomonal infections [14, 45, 46]. Low resistance to gentamicin was also reported in two studies by Teixeira et al. [47] and Asghar and Ahmed [16]. In Italy, a resistance-decreasing trend was observed for amikacin (11.7%) and tobramycin (21.1%) during the 2020–2022 era [48]. Also, Mesquita et al. [49] detected lower resistance to amikacin (8.8%) and gentamicin (19.5%) during the pandemic in Brazil.

The current study used EPI to investigate the efflux pump overexpression phenotypically. Targeting the RND superfamily, EPI could be a significant adjunctive therapy for enhancing the effectiveness of antibiotics, decreasing resistance and attenuating Gram-negative pathogen virulence via pump inactivation. By using CCCP as EPI, 56.3% (18/32) and 58.8% (20/34) of FQ and aminoglycosides resistant isolates showed ≥ fourfold reduction in MIC for ciprofloxacin and gentamicin, respectively. This was in accordance with reports of Talebi-Taher and colleagues [50] and Venkataramana et al. [51] who had detected marked reduction in MIC in their isolates following CCCP addition. According to our observations, many *P. aeruginosa* isolates displaying a wide variety of resistant phenotypes exhibited greater susceptibility ciprofloxacin and gentamicin as makers of FQ and aminoglycosides in the presence of EPIs. These findings verified the efflux systems' simultaneous multidrug extrusion [41, 50].

The frequency of PMQR genes within our FQ resistant isolates was 59.4% and that is comparatively higher than those detected in earlier investigations performed on the same microorganism [27, 52]. Qnr frequencies in current study were found to be 37.5%, 31.2% and 12.5% for *qnrS*, *qnrA* and *qnrB*, respectively. These findings are higher than those of previous Egyptian study which reported the existence of *qnrS* and *qnrB* genes in 2.7% and 1.8% of *Pseudomonas spp* [53]; while during the pandemic, the prevalence increased to 15% and 10%, respectively [54]. El-Badawy et al. [26] documented high frequency of *qnrS* gene (79.5%), while none of their isolates were carrying *qnrA* or *qnrB* genes. In China, *qnrA* gene* was* found in single isolate from 256 *P. aeruginosa* clinical isolates [55]. In several prior investigations, *Qnr* determinants were not detected in all *P. aeruginosa* isolate [56, 57].

Two or more PMQR genes were detected in 34.4% of our isolates. These outcomes were consistent with that of Ali et al. [52] and lower than El-Badawy et al. [26] and Al-Marjani [58]. The co-occurrence of many PMQR genes may be the cause of the elevated FQ resistance rates, and the horizontal transmission of quinolone resistance resulting in fewer therapeutic options for treating *Pseudomonas* infections.

*P. aeruginosa* resistance to aminoglycoside is often associated with the production of different AMEs [43] so, the increased incidence of these enzymes is a serious issue. In this study, 94.1% of the resistant strains carried AMEs-encoding genes. This result matched with that reported by Panahi et al. (96.2%) [59], but differed from that of Asghar and Ahmed (46.1%) [16]. Moreover, Ahmadian et al. [43] mentioned lower prevalence of AMEs-coding genes in *P. aeruginosa* (79%). Further, our findings counter data from the USA which reported the minor role of AMEs in bacterial resistance to aminoglycoside [60].

PCR findings showed that *aac(6’)-Ib* (79.4%), *aph(3″)-Ib* (67.7%) and *aac(3)-Ia* (35.3%) were more prevalent than *ant(2″)-Ia* (29.4%), while* ant(4’)-IIa* was not detected in any of the investigated isolates. The higher *aac(6’)-Ib* prevalence in this study was also detected in earlier research from Venezuela [47] and Iran [43, 61], while it was significantly greater than the results obtained by El-Far et al. [44]. During Covid-19 outbreak, Afify et al. [54] revealed the presence of *aac(6’)-Ib* and *ant(2″)-Ia,* respectively, in 40% and 50% of the screened *P. aeruginosa* isolates in Egypt. Another different results were obtained by Michalska et al. [27] who reported that the most commonly identified gene was *ant(2″)-Ia* (36.0%). Compared to prior years, the frequency of these genes in our results showed a rising trend of resistance.

Remarkably, individual aminoglycoside resistant strains harbored two to four AMEs genes. In our study, *aac(6’)-Ib* and *aph(3″)-Ib* were collectively found in 14.7% of the isolates, while 8.8% of isolates contained both *aac(6’)-Ib* and* ant(2″)-Ia*. Additionally, 23.5% of the isolate carried three genes. These results were similar to what observed by Hazra and co-workers [14], but differed from those of Vaziri et al. [62].

Concerning aminoglycoside resistance methyltransferase gene, PCR assays indicated the existence of RMT genes in 55.9% of the isolates with *rmtB* accounting for the majority (32.3%), followed by *rmtD* (17.6%) and *armA* (14.7%)*.* That rate of *rmtB* was lower than that detected in Brazil (44.4%) [63] and Theodor Bilharz Research Institute in Egypt (51.5%) [44], but much higher than those from Saudi Arabia, where *rmtB* and *armA* were found in 7.6% and 1.5% of isolates, respectively [16]. In a Chinese research, *armA* was found in 22% of *P. aeruginosa* strains [64], while in the study of Kashfi et al. [45], *armA* was found in 55% of isolates recovered from burn patients. This variation in the frequency of resistance genes may be explained by the differences in prescription patterns of aminoglycoside or regional disparities in the incidence of genes responsible for resistance to aminoglycoside.

All FQ resistant strains included in our study harbored both of *gyrA* and *parC* genes because they are continuously produced at a relatively steady rate and their absence results in cell death [65]. This finding align with that mentioned previously [66]. The DNA sequences of the resistant strains and the reference *P. aeruginosa* ATCC27853 were compared and no mutation was detected in both *gyrA* and *parC* genes. Different results obtained from other research which reported that the main pathway of FQ resistance among clinical isolates of *P. aeruginosa* is mediated primarily through mutations in *gyrA* as a main target for fluoroquinolones and additional mutations in *parC* my result in elevated level of quinolone resistance [66–68]. Gorgani et al. [69] demonstrated that the presence of *gyrA* mutations does not always indicate FQ, resistance, but it raises the likelihood of developing resistance to them.. Additionally, Nouri et al. [68] mentioned the significance of other resistance mechanisms (like innate membrane impermeability, *gyrB, parE* alterations or active efflux pumps) in the level of FQ resistance in *P. aeruginosa* isolates. This could explain the minor role of QRDRs mutations in FQ resistance observed in our study.

The most well-characterized efflux pump systems linked to antibiotic resistance are MexAB-OprM, MexCD-OprJ, MexEF-OprN, and MexXY-OprM [30]. In our study, 96.9% of FQ resistant isolates displayed overexpression of at least one of the four tested genes with *mexA* predominating (87.5%) followed by *mexC* (34.4%), whereas *mexE* (12.5%) was the least one. Only 9.4% of these isolates showed overexpression for all tested efflux pump genes. *MexY* were overexpressed in 31.5% of FQ and aminoglycosides resistant strains. These results were similar to that obtained by Okasha [33] who reported that 89.1% of their FQ resistant strains upregulated one or more of the *mex* genes, but differed from them in the percentage of isolates that overexpressed all tested RND efflux genes (27.4%). Al Rashed et al. [4] reported lower results (8%). Moreover the over expression of *mexA* in our isolates was much higher than that detected in Iran by Pourakbari et al. (55.5%) [70]. A cross sectional study made by Kishk et al. [71] in Suez Canal University Hospital showed that efflux pump overexpression could be the primary cause of bacterial multi-drug resistance as 88.2% of their *P. aeruginosa* strains overexpressed *mexA* gene, this is concurring with our results.

Considering the statistical analysis, no significant association was found between *mexY* over-expression level and resistance to both of FQ and aminoglycosides. Also, no significant correlation was found between the upregulation of *mex genes* and resistance to levofloxacin and gentamicin (*P* > 0.05). In the same manner, non-significant association was found between *mex* genes hyperproduction and β-lactams resistance in *P. aeruginosa* obtained from lower respiratory tract infections in Turkey [72]. In contrast, Zahedi Bialvaei et al*.* [41] observed a significant relationship between overexpression of *mexB* or *mexY* genes and resistance to different antibiotics with the exception of colistin.

Regarding agreement between genotypic and phenotypic techniques employed in the study, 87.1% of FQ resistant strains carrying efflux pump genes showed phenotypic efflux hyper-production. Abdallah et al. [73] concurred with our findings and documented that 91.3% of *P. aeruginosa* strains harboring efflux genes were phenotypically positive. In addition, Abdel Khalek et al. [74] detected 100% correlation between genotypic and phenotypic methods. However, Talebi-Taher et al. [50] observed a lower frequency as all of their *P aeruginosa* strains overexpressed *mexA* gene but around half of them had an overexpressing phenotype and this was ascribed to inactive pumps that might eventually become overexpressed.

In the current investigation, 24 distinct RAPD clusters were found among 38 resistant isolates (63.2% of polymorphisms). However, cross-infection is possible to occur in hospitals, indicating a problem with nosocomial infection management.

## Conclusions

The COVID-19 pandemic resulted in the uncontrolled use of broad-spectrum antibiotics, which raised the level of antibiotic resistance. Our findings demonstrated significant resistance to crucial antimicrobial drugs like fluoroquinolones and aminoglycosides, which are the preferred antibiotics recommended for management of *P. aeruginosa* infections. Efflux pump genes overexpression and enzymatic modification of aminoglycosides were the most prevalent resistance mechanisms found our isolates. Phylogenetic results revealed the probability of cross contamination so, proper infection control measures and periodic monitoring for antibiotic resistance are necessary to limit further dissemination and maintain efficacy of these essential antibiotic classes.

## Data Availability

The nucleotide sequences of gyrA and ParC genes were deposited into GenBank with accession numbers from PP544821 to PP544852 for gyrA and from PP544853 to PP544884 for parC. The other datasets used in this study are available from the corresponding author on reasonable request.

## Abbreviations

AACs: Aminoglycoside acetyltransferases
AMEs: Aminoglycoside-modifying enzymes
ANTs: Aminoglycoside nucleotidyl transferases
APHs: Aminoglycoside phosphotransferases
CCCP: Carbonyl cyanide 3-chlorophenyl hydrazine
CLSI: Clinical and Laboratory Standards Institute
EPI: Efflux pump inhibitor
FQ: Fluoroquinolones
LB broth: Luria–Bertani broth
MDR: Multidrug-resistant
MHA: Mueller-Hinton agar
MIC: Minimum inhibitory concentration
PDR: Pan drug-resistant
PMQR: Plasmid-mediated quinolone resistance
QRDRs: Quinolone-resistance determining regions
RAPD: Random amplified polymorphic DNA
RMT: Resistance methyltransferase
RND: Resistance–nodulation–division
SPSS: Statistical Package of Social Science
XDR: Extensively drug-resistant
